# ZnO-PLLA Nanofiber Nanocomposite for Continuous Flow Mode Purification of Water from Cr(VI)

**DOI:** 10.1155/2015/687094

**Published:** 2015-11-23

**Authors:** T. Burks, F. Akthar, M. Saleemi, M. Avila, Y. Kiros

**Affiliations:** ^1^Department of Chemical Engineering and Technology, Royal Institute of Technology (KTH), 100 44 Stockholm, Sweden; ^2^Department of Engineering Sciences and Mathematics, Luleå University of Technology, 971 87 Luleå, Sweden; ^3^Division of Functional Materials, Royal Institute of Technology (KTH), 164 40 Kista, Sweden; ^4^Department of Materials and Environmental Chemistry, Arrhenius Laboratory, Stockholm University, 10691 Stockholm, Sweden

## Abstract

Nanomaterials of ZnO-PLLA nanofibers have been used for the adsorption of Cr(VI) as a prime step for the purification of water. The fabrication and application of the flexible ZnO-PLLA nanofiber nanocomposite as functional materials in this well-developed architecture have been achieved by growing ZnO nanorod arrays by chemical bath deposition on synthesized electrospun poly-L-lactide nanofibers. The nanocomposite material has been tested for the removal and regeneration of Cr(IV) in aqueous solution under a “continuous flow mode” by studying the effects of pH, contact time, and desorption steps. The adsorption of Cr(VI) species in solution was greatly dependent upon pH. SEM micrographs confirmed the successful fabrication of the ZnO-PLLA nanofiber nanocomposite. The adsorption and desorption of Cr(VI) species were more likely due to the electrostatic interaction between ZnO and Cr(VI) ions as a function of pH. The adsorption and desorption experiments utilizing the ZnO-PLLA nanofiber nanocomposite have appeared to be an effective nanocomposite in the removal and regeneration of Cr(VI) species.

## 1. Introduction

Continuous increase in population and industries has led to elevated releases of toxic substances into the environment. These substances seriously influence the metabolism of living organisms that can cause permanent if not lethal damage, subsequently, placing a potential hazard to the ecosystem. Global environmental organizations, such as, the Environmental Protection Agency (EPA), have made the removal of heavy metal contaminates including chromium and other toxic substances from aquatic systems an important issue for environmental engineering and other technical areas. Moreover, there is a need for the development of efficient and cost effective processes for the treatment of discharge streams and recovery of valuable components [1, 2].

As an inorganic contaminant, the transitional metal, chromium, exists in the oxidation states Cr(III) and Cr(VI). Cr(VI) is considered as a grave concern for water pollution [3]. Chromium is a metal found in natural deposits as ores containing other elements. It is most often produced by industrial processes such as electroplating, stainless steel, protective coatings on metal, magnetic tapes, and pigments for paints, cement, paper, and rubber, as a composition of floor covering and other materials [4–8]. As a result of industrial and waste disposal locations, chromium has been released to the environment via leakage and poor storage during manufacturing or improper disposal practices [9, 10]. Due to the elemental nature of chromium and its solubility and mobility characteristics in the environment [11], the Environmental Protection Agency (EPA) has set an enforceable standard level called a Maximum Contaminant Level (MCL) for chromium, which is designated as 0.1 mg L^−1^. Hexavalent chromium is a toxic element having severe outcome on public health which is considered to be a mutagen, teratogen, and carcinogen and whose exposure, ingestion, or inhalation may also cause respiratory failure and ulcerations of the body [12–14]. Cr(III) exists predominantly as Cr^+3^ below pH 3.5. Cr(III) exhibits lower toxicity and is immobile from alkaline to slightly acidic conditions. Drinking water that contains more than 0.05 mg L^−1^ chromium is considered to be very toxic for living beings [11]. Hence, the effective removal or reduction of Cr(VI) to Cr(III) from contaminated water and wastewater is very important [15].

Metal oxides such as ZnO are essential for the development of efficient and smart materials [16]. ZnO nanoparticles can be found in vast applications including pharmaceutical and cosmetic [17], rubber manufacture [18], and electronic materials [19]. Other conventional and biomass derived adsorbents that have been used in the removal of heavy metals are activated carbon [20], wheat bran [21], activated neem leaf powder [22], mussel shell [23] and walnut shell powder [24], and peanut shells [25].

Depending upon their particle size and concentration, there are some advantages for using the aforementioned nonconventional adsorbents; they can remove up to 90% of the adsorbent. The adsorbents are also readily available and require less maintenance and supervision. On the other hand, they do require further treatment before they can be used to extract the heavy metal of choice [26]. The advantages and interesting properties of ZnO such as low cost and UV-blocking render them being a very good candidate for the adsorption studies [27]. Since the past decade, there has been an increased amount of activity regarding the toxicity of metal oxide nanoparticles and other bulk materials as to how they interact with both living and nonliving organisms. Baruah et al. illustrated using commercially available nonwoven polyethylene microfibers as a substrate material where ZnO nanorods were grown by a method called chemical bath deposition [28]. Moreover, Rodríguez-Tobías et al. fabricated antibacterial poly(D,L-lactide) nanofibers impregnated with zinc oxide nanoparticles to investigate the morphological, mechanical, and antibacterial properties [29]. Capitalizing upon this idea of fibrous substrates, from research, it was found that nanofibers are an exciting new class of material with diameters ranging from a few nanometers up to micrometers [5, 30]. Also, there are other characteristics as high surface area to volume ratio, tensile strength, and flexibility that make these fibers an excellent candidate for applications that encompasses surface reactions to take place such as separation/filtration membranes and surface-supported chemical reactions [31, 32]. These fibers are typically on an order of magnitude of two or three times smaller than fibers conventionally produced. The electrospinning technique was employed in the preparation and formation of the polymer nanofibers in the form of a nonwoven mat. This technique is a simple and versatile method of producing a fairly large-scale amount of fibers from a variety of materials. These fibers, which are nonmechanical and electrostatic, involve the utilization of a rather high electrostatic field to charge polymer solution's surface droplet, thus inducing the ejection of a liquid jet to be deposited onto a collector's plate.

In order to utilize these materials, we report the fabrication of highly flexible nanofibers of poly-L-lactide (PLLA) by electrospinning, which was used as a substrate for the growth of radially oriented ZnO nanowires by the chemical bath deposition method. Assembled nanostructured polymeric nanofibrous mat retains the flexibility and high surface area; it has the additional functionality of ZnO nanorods. As a direct result of this assembled nanostructure, we devised a simple setup for adsorption and desorption of Cr(VI) ions from the single metal aqueous solution in a continuous flow mode.

## 2. Experimental

### 2.1. Materials

Poly-L-lactide (Mw = 100000), chloroform, sodium hydroxide, and hydrochloric acid were purchased from Sigma-Aldrich. All chemicals were analytical grade reagents and used as received without further purification. High purity water with a resistivity of 18 MΩ cm was used throughout all the experiments.

### 2.2. Fabrication of Poly-L-Lactide (PLLA) Flexible Nanofibrous Mat

Poly-L-lactide nanofibers were produced by a technique known as electrospinning. Chloroform was used to dissolve the polymer (7 wt%) while stirring for 24 h. The sample was loaded into a syringe composed of a stainless steel needle (0.8 mm in diameter). The syringe was connected to the anode of voltage supply (Brandenburg). The collector's plate was covered with aluminum foil and was connected with the cathode of a voltage supply. A voltage of 9-10 kV was applied between the needle and the collector. The needle containing the polymer solution was mounted to a syringe pump (Cole-Parmer) in a horizontal direction. The distance from the tip of the needle to the collector is 10 cm with a flow rate of 1.0 mL h^−1^. Bestowing high voltage power supply, the potential was applied to the hypodermic needle (Needle Nokor Admix) on the end of the syringe.

### 2.3. Synthesis of ZnO-PLLA Assembled Nanostructure

For a total time of 30 min, the PLLA nanofibers were immersed into colloidal ZnO suspension prepared by modifying a method described by Bahnemann et al. [33], subsequently allowing it to dry. The “seeded” nanofibers were immersed into a mixed aqueous solution of 20 mM each of Zn(NO_3_)_2_ and hexamine and heated to 75°C for 6 h. The prepared PLLA-ZnO assembled nanostructured material was washed followed by drying in a vacuum oven for 1 h.

### 2.4. Continuous Flow Processing for Cr(VI) Adsorption

The PLLA-ZnO assembled nanostructured material so prepared was inserted into a glass tube. This column was connected to a 100 mL glass bottle (reservoir) via a peristaltic pump. The adsorption experiments were performed by the mixing of K_2_Cr_2_O_7_ in aqueous solution with 0.01 M NaNO_3_ and pumped at a flow rate of 2.0 mL min^−1^ and total time of 1500 minutes. The solution was continuously supplied onto the PLLA-ZnO assembled nanostructure. After each designated time, a sample was taken from the reservoir and prepared for analysis. The amount of Cr(VI) ions in the supernatant was determined with the Inductively Coupled Plasma (ICP-OES iCAP 6000 Series Thermo Scientific).

In the aqueous phase, the amount of Cr(VI) adsorbed per unit mass of adsorbent *q*_*e*_ (mg g^−1^) was calculated using

(1) $$\begin{array}{c} q_{e}=\frac{\left(C_{0}-C_{e}\right)V}{m}, \end{array}$$

where *C*_0_ is the initial chromium concentration (mg L^−1^) and *C*_*e*_ is the chromium concentration in the aqueous phase at equilibrium (mg L^−1^). *V* is the total aqueous volume (L) and *m* is the mass of the ZnO-PLLA nanofibers (g).

For the desorption experiment, the Cr(VI) solution was mixed with NaOH solution at pH = 9. The amount of Cr(VI) released from the ZnO nanorod-PLLA nanofiber into the supernatant was determined. The recovery efficiency (*R*) of Cr(VI) is calculated as

(2) $$\begin{array}{c} R\left(\;\%\;\right)=\frac{C_{des}}{C_{ads}}\times 100, \end{array}$$

where *C*_des_ is the amount of Cr(VI) released into the solution and *C*_ads_ is the amount of Cr(VI) adsorbed onto the ZnO-PLLA nanocomposite.

## 3. Results and Discussion

### 3.1. Characterization of PLLA and ZnO-PLLA Nanofibers

**Figure 1:**
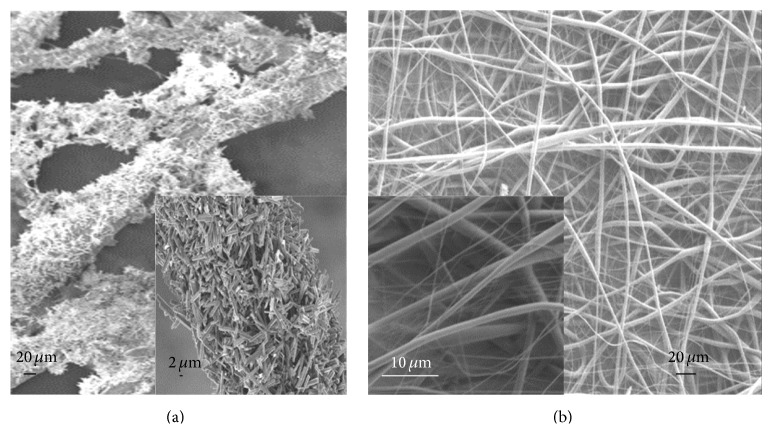
(a) SEM micrographs of the assembled ZnO-electrospun PLLA nanofibers. Inset: representation of the assembled nanostructure. (b) SEM micrographs of electrospun PLLA nanofibers. Inset: high resolution cross section of PLLA nanofiber.

Highly flexible nanofiber nanocomposite has been prepared using a combination of various chemical techniques. Sugunan et al. reported that the growth of ZnO nanorods is dependent upon a set of parameters and growth conditions such as concentration and temperature, which directly affect the diameter and length of the nanofibers [34]. Morphology and microstructure of the nanocomposite have been investigated by scanning electron microscopy (SEM).  Figure 1(a) presents a micrograph of electrospun PLLA nanofibers and Figure 1(b) shows these nanofibers with assembled ZnO. It can be observed in Figure 1 that the growth of ZnO nanorods and fabrication of PLLA nanofibers are intact and uniform without degradation. The cross section of PLLA nanofibers ranged from few tens of nanometers to 4 *μ*m. Moreover, the cross section of the nanocomposite has increased up to 12 *μ*m, which is dependent on the parameters such as growth conditions and physiochemical properties (temperature and concentration) of ZnO nanorods.

### 3.2. Effect of pH

**Figure 2:**
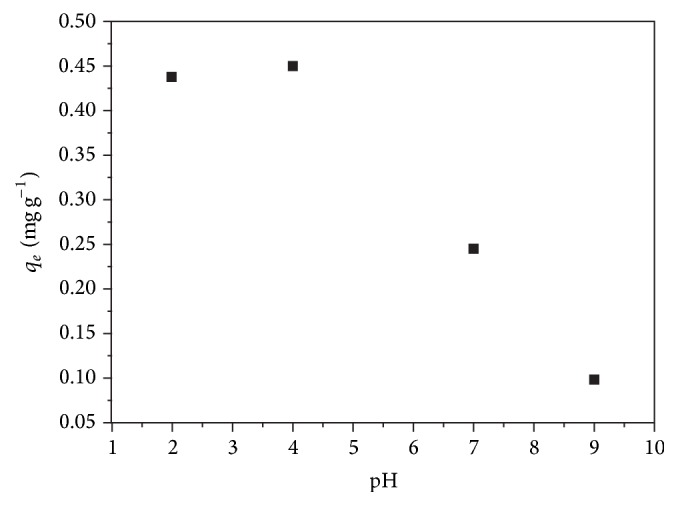
pH dependence, Cr(VI) initial concentration = 2.3 mg L^−1^, contact time = 24 h, total volume = 100 mL, and flow rate = 2 mL min^−1^.

One parameter that can have a consequential impact on the adsorption of Cr(VI) is pH. The uptake of Cr(VI) by this highly flexible nanofiber nanocomposite from solutions was studied as a function of contact time, pH, and Cr(VI) concentration. The oxidation states of chromium range from −2 to +6; in nature, it is mainly found in two oxidation states Cr(III) and Cr(VI). Cr(III) and Cr(VI) differ in many of their properties such as pH, charge, physiochemical characteristics, mobility, and toxicity. Cr(VI) speciation exists in solution as chromate (CrO_4_^2−^), bichromate (HCrO_4_^2−^), and dichromate (Cr_2_O_7_^2−^) [3, 4]. In this study, Cr(VI) was the only species in the solution considered as our starting material which was K_2_Cr_2_O_7_. For each sample, 2-3 wavelengths were selected to ensure sensitivity, improved precision, and detection limits while maintaining minimal interference from other elements is not accounted. The axial plasma orientation was used for the analysis of Cr(VI) ions in solutions. The ICP standard solutions were prepared and a calibration curve was generated. The weight of the metal oxide nanoparticles was calculated from the obtained ICP analysis. The amount of Cr(VI) ions in the supernatant was determined. The amount of Cr(VI) adsorbed on the flexible nanofiber nanocomposite platform as a function of pH in the range of 2 to 9 was experimentally determined and the data are shown in Figure 2. The optimum pH for the adsorption of Cr(VI) species by the flexible ZnO-PLLA nanofiber nanocomposite is ≤3.5. At low pH, better adsorption of Cr(VI) was achieved which may be attributed to the presence of large amount of hydrogen ions (H^+^), as also reported in the literature [5, 35]. The adsorption of Cr(VI) in the acidic pH range is similar to the work of Avila et al. who investigated the adsorption of Cr(VI) using surface functional polyacrylonitrile (PAN) nanofibers at pH = 2 [36].

The pH level can neutralize the negatively charged ZnO adsorbent surface, subsequently reducing the hindrance to the diffusion of Cr(VI) anions. Conversely, at increased pH values, the abundance of hydroxide ions (OH^−^) increases hindrance to diffusion of Cr(VI) anions, thereby reducing the removal efficiency. In alkaline solution, the surface charge of ZnO is negative. With such chromium species as neutral H_2_CrO_4_ and negatively charged HCrO_4_^−^, CrO_4_^2−^ and Cr_2_O_7_^2−^ would electrostatically be repelled. Therefore, at higher pH values, the adsorption of Cr(VI) would decrease [37].

### 3.3. Influence of Zeta Potential on ZnO

**Figure 3:**
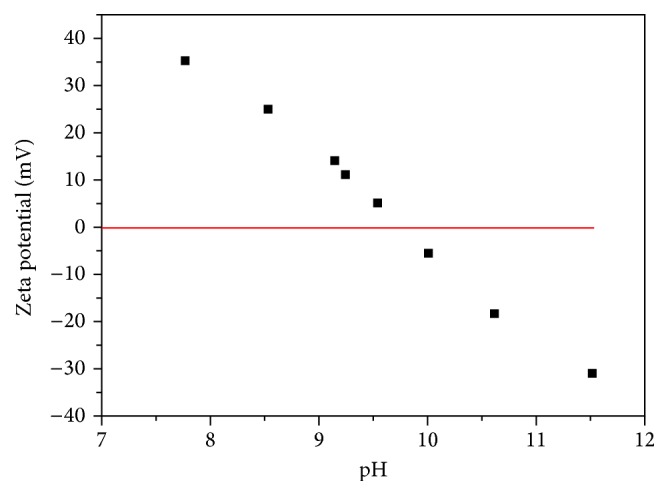
Zeta potential of ZnO nanostructures.

The main goal of performing zeta potential is to gain understanding on the dispersion behavior of the material. As illustrated in Figure 3, there is a change in zeta potential as the ZnO nanoparticles are titrated against NaOH as the point of zero charge (PZC) for ZnO is being determined. It can be observed that as the ZnO reaches the PZC, the surface charge of the oxide becomes neutral. Subsequently, very little to no adsorption of the Cr(VI) was obtained. As the pH of the metal oxide approaches the acidic regime, the predominant form of Cr(VI), as an ion, is HCrO_4_^−^; therefore, the surface becomes more positively charged, where the maximum adsorption of Cr(VI) in solution can occur. Other studies have reported that the adsorption of the zinc species affects the sign and the magnitude of the surface charge of the zinc oxide nanoparticles [38, 39]. At higher positive PZC, the dominant species were reported to be Zn^2+^ [40].

### 3.4. Effect of Contact Time on ZnO-PLLA Nanofibers

In order to assess the optimal contact time for the adsorption of Cr(VI) species, the experiments were performed at pH = 3.5, which was found to be the ultimate pH for this study as shown in Figure 2. Choudhury et al. reported that the rapid adsorption of metals on nanoparticle surface was due to the external surface adsorption [41]. Several batch experiments were performed in order to determine the time required to reach equilibrium. After a certain period, the adsorption curve reached a plateau region where no more Cr(VI) species were adsorbed. Based on this observation, the optimum contact time for adsorption of Cr(VI) species at this pH value was determined to be 1300 minutes.

**Figure 4:**
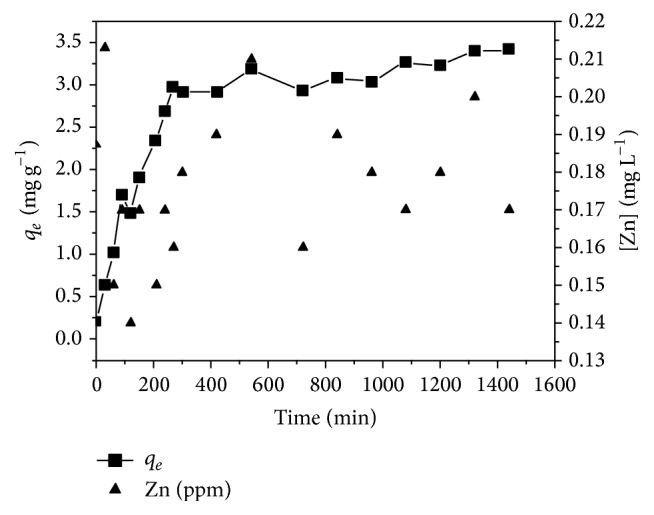
Effect of Contact Time: Cr(VI) initial concentration = 2.3 mg L^−1^, initial solution pH = 3.5, total volume = 100 mL, and flow rate = 2 mL min^−1^.

Figure 4 illustrates the effect on the amount of Cr(VI) adsorbed as a function of time. The equilibrium can be obtained when the maximum interaction of the ZnO-PLLA nanofiber nanocomposite occurred at a time of 1300 minutes and initial pH of 3.5 resulting in maximum adsorption of 3.5 mg g^−1^ of Cr(VI) adsorbed. For the Cr(VI) species, at acidic pH the (HCrO_4_^−^) species has been known to be dominant. It is important to note that the guideline value, derived from tolerable daily intake, daily consumption of water, and body weight, is 0.05 mg L^−1^ [42]. Thus, methods for drinking water management by means of adsorption ZnO-PLLA nanofibers in the small concentration ranges have appeared to be effective.

As shown in Figure 2, the PZC for ZnO nanostructures was found to be 10. The zeta potential was highly influenced by pH. The surface charge on ZnO was increased positively as the pH became more acidic. When ZnO is introduced into solutions composed of negative species, adsorption can readily occur. As the zeta potential curve moves toward higher pH, the charge density changes such that the electrostatic interaction is no longer applicable; instead the system within solution shows more of a repulsive scenario. The effect of repulsive interaction can be most beneficial in systems, where the interest is regeneration of the nanomaterial to be reused for several cycles of adsorption and desorption operations. As the ZnO is attached to the PLLA nanofiber, the amount of Zn species dissolved in the solution was found to be less than 0.25 mg L^−1^, showing a stable performance for adsorption.

### 3.5. Desorption of ZnO-PLLA Nanofibers

**Figure 5:**
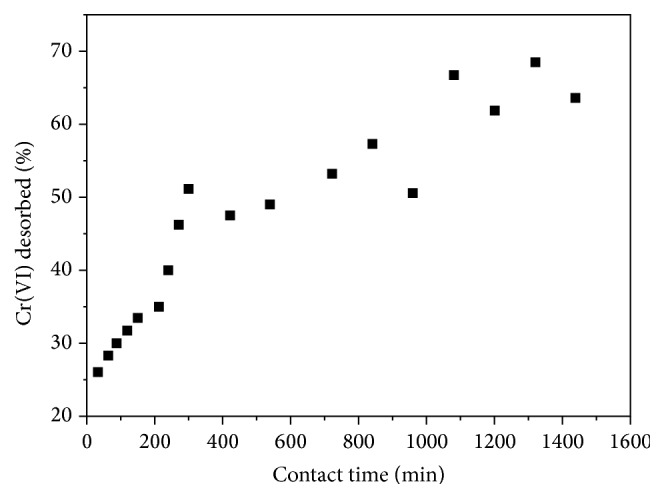
Desorption of Cr(VI) from loaded nanofiber: concentration of NaOH = 0.01 M, total volume = 100 mL, and flow rate = 2 mL min^−1^.

To regenerate the adsorbent nanocomposite nanofiber platform, it is essential that all adsorbed Cr(VI) species are desorbed so that the platform can be reused as this step is crucial for recovery of the species for recycling in other processes. It also can give rise to information regarding whether Cr(VI) is reversibly or irreversibly adsorbed onto the ZnO-PLLA nanocomposite. The recovery of Cr(VI) species from ZnO-PLLA nanofiber nanocomposite was tested at pH = 12. In the alkaline regime, the OH^−^ ions interact with the Cr(VI) ions attached to the platform thereby releasing the Cr(VI) into solution, thereby indicating that the desorption process was reversible.  Figure 5 shows that the maximum recovery of Cr(VI) from the ZnO-PLLA nanofiber nanocomposite into solution was 60%.

This could be attributed to the fact that the sites on the material are initially occupied with Cr(VI) and when introduced to pH of 12 the Cr(VI) ions leave the sites and are released back into solution [43]. Also, in the alkaline regime, the surface charge of ZnO is negative. Metal recovery and reuse of the adsorbent can become economically viable in the overall process [44].

## 4. Conclusion

In this work, PLLA and ZnO-PLLA nanofiber nanocomposite were successfully fabricated and have been shown to be an effective material in the removal of highly toxic Cr(VI) species. Given the optimum adsorption conditions, the adsorption equilibrium can be reached after several hours. The removal of Cr(VI) using the nanocomposite was highly dependent on pH. At pH < 3.5, the maximum adsorption of Cr(VI) was obtained. Electrostatic and surface attractions between ZnO and Cr(VI) species control the interactions between the flexible nanocomposite platform and the Cr(VI) species. In the basic pH range solution, Cr(VI) can be regenerated and the ZnO-PLLA nanofiber nanocomposite can be reused. A 60% desorption efficiency of Cr(VI) was accomplished using NaOH. The ZnO-PLLA nanofiber nanocomposite has shown great potential in applications such as water and wastewater treatment for the removal of heavy metal ions of Cr(VI) species.
